# Sex steroids, growth factors and mammographic density: a cross-sectional study of UK postmenopausal Caucasian and Afro-Caribbean women

**DOI:** 10.1186/bcr2325

**Published:** 2009-06-22

**Authors:** Valerie A McCormack, Mitch Dowsett, Elizabeth Folkerd, Nichola Johnson, Claire Palles, Ben Coupland, Jeff M Holly, Sarah J Vinnicombe, Nicholas M Perry, Isabel dos Santos Silva

**Affiliations:** 1 Cancer Research UK Epidemiology and Genetics Group, Department of Epidemiology and Population Health, London School of Hygiene & Tropical Medicine, Keppel Street, London WC1E 7HT, UK; 2 International Agency for Research on Cancer, 150 cours Albert Thomas, Lyon 69008, France; 3 The Academic Department of Biochemistry, The Royal Marsden Hospital, Fulham Road, London SW3 6JJ, UK; 4 Cancer Research UK Epidemiology and Genetics Group, Breakthrough Breast Cancer Research Centre, Institute of Cancer Research, 237 Fulham Road, London SW3 6JB, UK; 5 University Department of Clinical Science at North Bristol, Southmead Hospital, Southmead Road, Westbury-on-Trym, Bristol BS10 5NB, UK; 6 Breast Assessment Centre, St Bartholomew's Hospital, Barts and The London NHS Trust, West Smithfield, London EC1A 7BE, UK

## Abstract

**Introduction:**

Sex steroids, insulin-like growth factors (IGFs) and prolactin are breast cancer risk factors but whether their effects are mediated through mammographic density, one of the strongest risk factors for breast cancer, is unknown. If such a hormonal basis of mammographic density exists, hormones may underlie ethnic differences in both mammographic density and breast cancer incidence rates.

**Methods:**

In a cross-sectional study of 270 postmenopausal Caucasian and Afro-Caribbean women attending a population-based breast screening service in London, UK, we investigated whether plasma biomarkers (oestradiol, oestrone, sex hormone binding globulin (SHBG), testosterone, prolactin, leptin, IGF-I, IGF-II and IGF binding protein 3 (IGFBP3)) were related to and explained ethnic differences in mammographic percent density, dense area and nondense area, measured in Cumulus using the threshold method.

**Results:**

Mean levels of oestrogens, leptin and IGF-I:IGFBP3 were higher whereas SHBG and IGF-II:IGFBP3 were lower in Afro-Caribbean women compared with Caucasian women after adjustment for higher mean body mass index (BMI) in the former group (by 3.2 kg/m^2 ^(95% confidence interval (CI): 1.8, 4.5)). Age-adjusted percent density was lower in Afro-Caribbean compared with Caucasian women by 5.4% (absolute difference), but was attenuated to 2.5% (95% CI: -0.2, 5.1) upon BMI adjustment. Despite ethnic differences in biomarkers and in percent density, strong ethnic-age-adjusted inverse associations of oestradiol, leptin and testosterone with percent density were completely attenuated upon adjustment for BMI. There were no associations of IGF-I, IGF-II or IGFBP3 with percent density or dense area. We found weak evidence that a twofold increase in prolactin and oestrone levels were associated, respectively, with an increase (by 1.7% (95% CI: -0.3, 3.7)) and a decrease (by 2.0% (95% CI: 0, 4.1)) in density after adjustment for BMI.

**Conclusions:**

These findings suggest that sex hormone and IGF levels are not associated with BMI-adjusted percent mammographic density in cross-sectional analyses of postmenopausal women and thus do not explain ethnic differences in density. Mammographic density may still, however, be influenced by much higher premenopausal hormone levels.

## Introduction

Mammographic density, the percentage of a mammogram that appears as radio-dense fibroglandular tissue, is one of the strongest markers of subsequent breast cancer risk [1]. This density may reflect an underlying process occurring within the breast that is causally related to breast cancer. Dense tissue is thought to occur as a consequence of higher rates of stromal and epithelial proliferation, factors that increase the risk of somatic mutations, epigenetic alterations and carcinogenesis [2]. Some endogenous sex steroids and growth factors are established breast cancer risk factors. Many of these are involved in epithelial cell proliferation and thus are potential drivers of the association between mammographic density and breast cancer risk. Candidate biomarkers are oestrogens, progesterone, testosterone, prolactin and premenopausal levels of insulin-like growth factor (IGF)-I [3-5]. Leptin may have a role in breast cancer development, especially in postmenopausal women where body mass index (BMI) is a breast cancer risk factor [6].

Two observations support a hormonal basis for mammographic density: density increases upon use of oestrogen and progestin hormonal therapies; and density is reduced by tamoxifen, a selective oestrogen receptor modulator [7,8]. Elucidating the biological processes that give rise to increased mammographic density might help identify ways in which this risk factor and ultimately breast cancer risk might be lowered. To date, however, studies that have examined the association between such biomarkers and density have revealed predominantly null or inconsistent associations. Their findings may have been limited by lack of adequate heterogeneity in biomarker or breast density distributions as most studies were conducted in relatively homogeneous populations. We try to overcome this by studying these associations in a heterogeneous multiethnic group of native and first-generation migrant women in the UK.

We have investigated mammographic density in relation to breast cancer biomarkers in an ethnically diverse study population. We recently reported that, consistent with their lower breast cancer risk nationally, the mean percentage mammographic density was lower in Afro-Caribbean women compared with Caucasian women [9] within our UK study population. This difference was largely explained by anthropometric and reproductive factors, whose influence on density may be mediated by plasma breast cancer biomarkers. Alternatively such biomarkers may be independently related to mammographic density [10-12]. We investigated these hypotheses here in a group of first-generation Afro-Caribbean women and native Caucasian women in the UK. The study aims were to assess: first, whether sex hormones, leptin, prolactin and IGFs are associated with mammographic density; second, whether there are ethnic variations in the distribution of these biomarkers; and third, if the first two aims hold true, whether variations in biomarker levels explain ethnic differences in mammographic density.

## Materials and methods

### Study population

We conducted a study of ethnic variations in mammographic density in the UK (as described elsewhere [9]). In brief, in 2005 and 2006 we randomly sampled an ethnically stratified group of women from the Central and East London Breast Screening Service who had undergone their second or further routine screening mammogram in 2004 at ages 50 to 65 years. The invitation to participate was mailed to women on average 1.5 years after their last mammogram, a strategy employed so as not to jeopardise breast screening uptake. In this population-based screening programme, three ethnic groups were included based on previously self-assigned ethnicity data: Caucasian (for those who selected 'White UK'), South Asian, and Afro-Caribbean ('African', 'Afro-Caribbean', 'Black African', Black-other' or 'Black Caribbean').

The randomly selected participants in each ethnic group were sent a questionnaire to self-complete, providing information on ethnicity, country of birth and known lifestyle breast cancer risk factors – that is, age at menarche, age at first full-term pregnancy, parity, total duration of breast feeding, past use of hormone therapy and oral contraceptives, alcohol use, smoking, height and weight – which were used to calculate the BMI as weight (kg)/height^2 ^(m^2^). Participants also provided consent for their mammograms to be digitised. Nonresponders were re-contacted (by post and by telephone) after 6 and 10 weeks to further increase participation rates.

After returning their questionnaire, postmenopausal Caucasian and Afro-Caribbean women not taking hormonal therapies were also asked to optionally have a 19 ml blood sample taken by their general practitioner, from which plasma and buffy coat was obtained and stored at -80°C. South Asian women were not asked to provide blood as in doing so we might have further compromised the very low response rate in this group [9]. The study was approved by the East London and The City Local Research Ethnics Committee.

With response rates of 59% and 41%, respectively, 267 Caucasian women and 213 Afro-Caribbean women participated in total. Of these women, 152 (56.9%) and 118 (55.3%), respectively, also provided a blood sample.

### Assays

Plasma levels of sex hormones were analysed at the Academic Department of Biochemistry, Royal Marsden Hospital Laboratories, London UK. Total oestradiol and oestrone were measured by an inhouse radioimmunoassay with a prior organic extraction. The lower detection limit was 3.0 pmol/l (no samples fell below this level). Testosterone was quantified using a coat-a-count method (Siemens Diagnostics formally known as DPC, Deerfield, IL, US) with a lower detection limit of 0.14 nmol/l (nine samples), sex hormone binging globulin (SHBG) was quantified using Spectria SHBG IRMA 68562 (Orion Diagnostica, Espoo, Finland) with a lower detection limit of 1.3 nmol/l (all samples were higher) and prolactin using DSL-4500 (Diagnostic Systems Laboratories, Webster, TX, US) with a lower detection limit of 27 mIU/l (all levels exceeded this). Leptin was measured using a solid-phase radioimmunoassay kit (DSL 23100i) from Beckman Coulter (High Wycombe, UK); the lower detection limit was 0.5 ng/ml and all levels were higher. Plasma IGF-I was measured using an ELISA assay (Diagnostic Systems Laboratories, Webster, TX, USA), IGF binding protein 3 (IGFBP3) was measured using a double-antibody radioimmunoassay, and IGF-II by an inhouse radioimmunoassay method (iodination of IGF-II) at the University of Bristol (laboratory of Prof. J Holly). Laboratory staff were blind to any identifiable data for the women. Free oestradiol was calculated from total oestradiol and SHBG [13]. Plasma levels less than the lower detection limit were assumed to have the value of this limit.

For sex hormones, 11 quality control samples (from one individual who was not a participant in this study) were included to assess repeatability. Intraclass correlation coefficients were 0.98 (95% confidence interval (CI): 0.97, 1.00) for oestradiol, 0.63 (95% CI: 0.31, 0.96) for oestrone, 0.80 (95% CI: 0.62, 0.98) for testosterone, 0.97 (95% CI: 0.95, 1.00) for SHBG and 0.67 (95% CI: 0.37, 0.96) for prolactin. Measurement errors for IGFs are also known to be low/moderate, with coefficients of variation of 6.6%, 12.0% and 3.9% for IGF-I, IGF-II and IGFBP3, respectively [14]. The IGF-I:IGFBP3 and IGF-II:IGFBP3 molar ratios were calculated by firstly converting IGF-I and IGF-II weights to molecular concentrations (dividing by their molecular weights of 0.13 and 0.025, respectively).

### Mammographic density

Density measurements from the 2004 breast screening round were analysed as this was the closest to the time of blood draw. All four films (cranio-caudal and medio-lateral oblique views for each breast) were digitised on an Array 2905 digitiser (optical density, 0 to 4.0; 75 μm; Array Corporation Europe, Roden, The Netherlands). The mammographic density was assessed by a single observer (VAM) using Cumulus, an interactive thresholding programme [15]. The nondense, dense and total breast areas in square centimetres, as well as the percentage mammographic density (100 × dense area/total area), were analysed. Films were read in randomly sorted batches of 200. One hundred films were independently re-read by the same observer and the reliability of a single density reading was 0.90.

### Statistical methods

We investigated whether ethnic variations existed in biomarker levels (second study aim) and then whether plasma biomarkers were associated with measures of mammographic density (first study aim) – and, if so, whether ethnic differences in these biomarkers (examined in the first stage) might explain ethnic variations in mammographic density (third study aim). Firstly, normal error regression models were used to assess the association of ethnic group (a binary indicator variable) with each biomarker, incorporating a natural logarithmic transformation of oestradiol, oestrone, SHBG, prolactin, testosterone, IGF-I, IGF-II and IGFBP3 to improve normality of residuals (second study aim). For these log-transformed biomarkers, their effects are thus in relative (percentage) terms. Adjustment for the laboratory batch (categorical variable) and age at blood collection (linear term) were included in these and all further models that included biomarkers. To avoid the over-influence of excessively raised biomarker levels in some women due to particular illnesses that might distort general associations, outliers were removed. Only prolactin had such an outlier, with a value of over 3,000 mIU/l (hyperprolactinaemia). The minimum detectable difference in mean biomarker levels between the two ethnic groups (n = 270) was 0.35 standard deviations (80% power, 5% false positive probability).

For the second and third study aims, where mammographic measures (percent density, dense area and nondense area) were the outcome, the average of the estimates from all four films on the square-root scale was calculated for each measure, providing a repeatable estimate of density (intraclass correlation coefficients of 0.97, 0.97, 0.98 and 0.99 for percent density, dense area, nondense area and total breast area, respectively). In these analyses, to provide more meaningful estimates of associations with density, effects of explanatory variables were referred to reference values of 16% density, and dense, nondense and total breast areas of 22, 118 and 140 cm^2^, respectively (median values in Caucasians, the reference group); the percent differences in density are thus differences on the absolute (not relative) scale of density measurement. We assessed whether biomarkers were related to mammographic density both using quintiles and using a linear trend of biomarker data (second study aim).

For all regression models, normal quintile plots and Cook's distances were examined to check the normality assumption and to identify outliers.

## Results

In total, 270 women provided blood samples. All 152 Caucasian women were born in the UK, and most of the 118 Afro-Caribbean women were born in the Caribbean (64%) or West Africa (30%) and came to the UK at a mean age of 20.9 years (standard deviation, 9.0). Within each ethnic group, women donors had similar characteristics to women who did not provide blood, with the exception of a higher participation rate among Afro-Caribbean women with a family history of breast cancer. Age at mammography ranged from 52 to 65 years and blood samples were taken on average 1.70 years later (95% reference range, 0.9 to 2.5 years), with no difference between ethnic groups (*t*-test *P *= 0.93). Breast cancer risk factor distributions in Afro-Caribbean women relative to Caucasian women suggest a more protective risk profile for most factors, characterised by later menarche, earlier age at first birth, higher parity, earlier menopause and lower use of exogenous hormones (Table 1). The major exception to this is BMI, for which the mean in Afro-Caribbean women was 3.2 kg/m^2 ^higher than that in Caucasian women (95% CI: 1.8, 4.5).

**Table 1:** Distributions of breast cancer risk factors and breast cancer biomarkers by ethnic group

	Caucasian women (n = 152)	Afro-Caribbean women (n = 118)
Lifestyle breast cancer risk factors^a^		
Age at mammography (years)	57.8 (3.3)	58.1 (3.6)
Age arrived in UK (years)	-	20.9 (9.0)
Age at menarche (years)	12.7 (1.5)	13.5 (2.0)
Percentage nulliparous	22.2	6.0
Age at first birth (years)	24.4 (5.8)	22.2 (5.2)
Age at menopause (years)	50.1 (4.8)	48.3 (5.4)
Body mass index (kg/m^2^)	26.2 (5.4)	29.4 (5.4)
Percentage previous hormone therapy use (ever)	35.5	24.6
Percentage previous oral contraceptive use (ever)	72.2	40.4
Percentage alcohol drinkers	86.1	57.4
Percentage current smokers	13.3	4.3
Percentage family history of breast cancer	17.8	11.9
Mammographic measures^b^		
Mammographic density (%)	16.2 (8.9 to 24.5)	8.3 (4.3 to 19.8)
Dense area (cm^2^)	21.2 (11.9 to 35.7)	17.3 (8.4 to 31.3)
Nondense area (cm^2^)	116.9 (86.7 to 160.0)	167.9 (115.3 to 235.9)
Total breast area (cm^2^)	141.5 (108.1 to 193.3)	195.2 (140.2 to 261.5)
Plasma biomarkers^c^		
Leptin (ng/ml)	12.3 (11.1, 13.5)^d^	19.1 (17.5, 20.7)^c^
Oestradiol (pmol/l)	21.7 (19.6, 23.9)	29.0 (25.6, 32.9)
Free oestradiol (pmol/l)	0.31 (0.27, 0.34)	0.44 (0.38, 0.50)
Oestrone (pmol/l)	72.9 (68.0, 78.2)	82.2 (74.9, 90.3)
Sex hormone binding globulin (nmol/l)	49.2 (46.1, 52.4)	42.5 (39.1, 46.2)
Testosterone (nmol/l)	0.95 (0.86, 1.05)	0.89 (0.78, 1.02)
Prolactin (mIU/l)	192 (177, 209)	193 (174, 213)
IGF-I (ng/ml)	144 (137, 152)	157 (146, 168)
IGF-I:IGFBP3 molar ratio	0.175 (0.166, 0.184)	0.199 (0.187, 0.213)
IGF-II (ng/ml)	896 (857, 938)	742 (698, 789)
IGF-II:IGFBP3 molar ratio	1.09 (1.04, 1.13)	0.94 (0.89, 1.00)
IGFBP3 (ng/ml)	4286 (4125, 4452)	4097 (3920, 4282)

IGF, insulin-like growth factor; IGFBP, IGF binding protein. ^a^Data presented as mean (standard deviation) or percentage. ^b^Data presented as median (25th to 75th percentiles). ^c^Data presented as geometric mean (95% confidence interval). ^d^Arithmetic mean (variable was not log transformed).

On average the percentage mammographic density was lower in Afro-Caribbean women compared with Caucasian women (median 8.3% compared with 16.2%; Table 1). This lower percent density is composed of a smaller absolute dense area (by 4 cm^2^) and a much larger nondense area, and, consequently, a much larger total breast area (difference in medians of over 50 cm^2^).

Mean plasma biomarker levels also revealed large ethnic differences (Table 1), which persisted after adjustment for processing batch and age (Table 2). Leptin, oestrogens, IGF-I and IGF-I:IGFBP3 molar ratios were all significantly higher amongst Afro-Caribbean women than Caucasian women; in particular, levels of oestradiol were 31% higher (95% CI: 12%, 52%) and levels of oestrone were 13% higher (95% CI: 1%, 27%). In the opposite direction, testosterone (although not statistically significant), SHBG, IGF-II and IGF-II:IGFBP3 molar ratios were lower by 6%, 14%, 17% and 14%, respectively, but there was no evidence of ethnic differences in IGFBP-3 or prolactin (Table 2).

**Table 2:** Comparison of plasma biomarkers and mammographic measures in Afro-Caribbean women compared with Caucasian women

	Adjustment^a^		
	
	Age and batch	Age, batch and BMI	Age, batch, BMI and breast cancer risk factors^b^
Plasma biomarker^c^			
Leptin (ng/ml)^d^	6.8 (4.8, 8.9)****	3.2 (1.7, 4.7)****	3.6 (1.9, 5.2)****
Oestradiol (pmol/l)	1.31 (1.12, 1.52)****	1.11 (0.96, 1.29)	1.11 (0.94, 1.31)
Free oestradiol (pmol/l)	1.39 (1.17, 1.65)****	1.11 (0.95, 1.30)	1.11 (0.94, 1.32)
Oestrone (pmol/l)	1.13 (1.01, 1.27)**	1.05 (0.93, 1.18)	1.05 (0.93, 1.19)
SHBG (nmol/l)	0.86 (0.78, 0.96)***	0.98 (0.89, 1.08)	0.96 (0.86, 1.07)
Testosterone (nmol/l)	0.94 (0.80, 1.11)	0.88 (0.74, 1.03)	0.86 (0.72, 1.03)
Prolactin (mIU/l)	1.02 (0.90, 1.16)	1.04 (0.91, 1.19)	1.00 (0.87, 1.16)
IGF-I (ng/ml)	1.09 (1.00, 1.19)**	1.13 (1.03, 1.24)***	1.12 (1.01, 1.24)**
IGF-I:IGFBP-3 molar ratio	1.13 (1.04, 1.23)***	1.20 (1.10, 1.30)***	1.20 (1.10, 1.32)***
IGF-II (ng/ml)	0.83 (0.77, 0.89)****	0.82 (0.76, 0.89)****	0.82 (0.76, 0.90)****
IGF-II:IGFBP-3 molar ratio	0.86 (0.80, 0.92)****	0.87 (0.81, 0.94)**	0.89 (0.82, 0.96)**
IGFBP-3 (ng/ml)	0.96 (0.91, 1.02)*	0.94 (0.89, 1.00)**	0.93 (0.87, 0.99)**
Mammographic measures^e^			
Mammographic density (%)	-5.4 (-7.8, -3.0)****	-2.5 (-5.1, 0.2)*	-0.8 (-3.7, 2.2)
Dense area (cm^2^)	-2.7 (-6.9, 1.4)	-1.9 (-6.4, 2.7)	0.7 (-4.4, 5.8)
Nondense area (cm^2^)	50.5 (33.2, 67.8)***	19.6 (7.7, 31.5)***	15.7 (3.1, 28.3)**
Total breast area (cm^2^)	48.7 (31.3, 66.0)****	18.7 (6.3, 31.1)***	17.0 (3.6, 30.5)**

IGF, insulin-like growth factor; IGFBP, IGF binding protein; SHBG, sex hormone binding globulin. ^a^Adjustment made for age at blood collection and processing batch (or age at mammography) in all models, in addition to listed factors. ^b^Age at menarche, parity, age at first birth, smoking, age at menopause. ^c^Data presented as ratio (95% confidence interval) of mean levels (Afro-Caribbean:Caucasian). ^d^Arithmetic scale (absolute differences reported as leptin did not need to be log-transformed). ^e^Data presented as difference (95% confidence interval) in means (Afro-Caribbean-Caucasian). *P < 0.1, **P < 0.05, ***P < 0.01, ****P < 0.001.

In an attempt to account for these differences, the lifestyle risk factors presented in Table 1 were examined as biomarker determinants (mutually adjusted for each other and for ethnicity), for which only the statistically significant associations are summarised here. BMI was strongly positively associated with levels of oestradiol (total and free), oestrone and leptin but was inversely associated with levels of SHBG. Both total and free oestradiol levels were lower in nulliparous compared with parous women, and among parous women they were lower in women who had a later age at first live birth. There was also weak evidence that free oestradiol levels were lower in women who had a later menarche, with levels estimated to be 4% lower for every 1-year delay in menarche (95% CI: -1%, 8%). There was no evidence that any other factors influenced oestrogen, SHBG or leptin levels. Testosterone levels were 34% higher (95% CI: 1%, 78%) in current smokers than in nonsmokers and there was weak evidence that the levels were higher in women with higher BMI. IGF-I levels were 17% lower (95% CI: 2%, 30%) in women with a BMI over 30 kg/m^2 ^compared with those with a BMI of under 22 kg/m^2^. We did not find any determinants of prolactin, IGF-II or IGFBP3.

As the distribution of the main determinants of biomarker levels (that is, BMI, age at first birth, age at menarche and smoking) also exhibit ethnic differentials (Table 1), the ethnic differences in biomarker levels were largely attenuated upon adjustment for these lifestyle risk factors and, in particular, for BMI (Table 2). Greatly increased leptin and oestradiol levels in Afro-Caribbean women compared with Caucasian women were partly accounted for by their higher mean BMI, although after full adjustment this ethnic group still had 3.6 ng/ml (95% CI: 1.9, 5.2) higher leptin levels and 11% (95% CI: -6%, 31%) higher oestradiol levels. Higher oestrone and lower testosterone and SHBG levels were entirely accounted for by differences in BMI distributions. In contrast, the ethnic differences in IGFs became larger after adjustment for lifestyle risk factors. Higher crude IGF-I and IGF-I:IGFBP3 levels in Afro-Caribbean women were negatively confounded by BMI, as BMI is higher in the Afro-Caribbean women but it is associated with lower IGF levels. After full adjustment, therefore, IGF-I:IGFBP3 molar ratios were 20% higher in Afro-Caribbean women (95% CI: 10%, 32%). IGF-II and IGFBP3 levels were lower in Afro-Caribbean women than in Caucasian women, and adjustment for BMI and other factors did not explain this difference.

The estimated effect of a twofold increase in each biomarker on percentage mammographic density and areas of dense and nondense tissue are presented in Table 3 (all tests of departures from linearity and of interactions with ethnicity were nonsignificant). Age-adjusted (at mammogram and blood collection), batch-adjusted and ethnicity-adjusted associations are firstly presented, and thereafter additional adjustment for BMI is made. Associations of leptin, total and free oestradiol and testosterone with percentage mammographic density showed similar patterns: before adjusting for BMI, there was a strong positive association with nondense area that was reflected in a strong inverse association with percent density, but no associations were observed with dense area. These associations were entirely explained by BMI. The percentage mammographic density was similarly lower in women with higher oestrone levels, but this association resulted not just from a larger nondense area but also from a smaller dense area. Even after adjustment for BMI, a twofold difference in oestrone levels was associated with a smaller dense area by 2.9 cm^2 ^(95% CI: -0.5, 6.3). The strong inverse association of BMI with SHBG was mirrored in the inverse association of SHBG with nondense area (with a corresponding association in the opposite direction with percent density), an association that was explained by BMI. Further adjustment for other breast cancer risk factors did not change the BMI-adjusted estimates substantially (data not shown).

**Table 3:** Difference in mammographic measures associated with a twofold increase in plasma biomarker levels

	Mammographic density (%)		Dense area (cm^2^)		Nondense area (cm^2^)	
	
	Adjustment 1^a^	Adjustment 2^b^	Adjustment 1^a^	Adjustment 2^b^	Adjustment 1^a^	Adjustment 2^b^
Leptin^c ^(per 9 ng/ml increase)	-4.2 (-5.6, 2.8)****	-1.2 (-3.4, 0.9)	-1.5 (-4.0, 1.0)	-0.6 (-4.3, 3.1)	41.0 (33.1, 49.0)****	10.1 (1.2, 18.9)**
Oestradiol	-3.1 (-4.5, -1.6)****	-1.2 (-2.8, 0.4)	-1.8 (-4.3, 0.7)	-1.3 (-4.1, 1.4)	22.4 (13.8, 31.1)****	2.9 (-3.6, 9.5)
Free oestradiol	-3.2 (-4.5, -1.8)****	-1.2 (-2.7, 0.4)	-1.9 (-4.1, 0.4)	-1.5 (-4.1, 1.1)	24.1 (16.4, 31.7)****	2.4 (-3.7, 8.6)
Oestrone	-3.9 (-5.9, -1.9)****	-2.0 (-4.1, 0.0)*	-3.6 (-6.8, -0.4)**	-2.9 (-6.3, 0.5)*	20.6 (8.4, 32.8)***	1.0 (-7.3, 9.4)
SHBG	4.1 (1.4, 6.9)***	0.2 (-2.4, 2.8)	2.3 (-1.7, 6.2)	1.5 (-2.9, 5.9)	-28.9 (-38.9, -18.9)****	0.2 (-9.3, 9.7)
Testosterone	-2.2 (-3.7, -0.7)***	-1.4 (-2.9, 0.1)*	-1.8 (-4.2, 0.6)	-1.7 (-4.1, 0.8)	11.3 (2.8, 19.7)***	3.4 (-2.5, 9.3)
Prolactin	2.2 (-0.1, 4.4)*	1.7 (-0.3, 3.7)*	1.8 (-1.5, 5.2)	1.7 (-1.6, 5.0)	-11.2 (-21.0, -1.4)**	-7.7 (-14.6, -0.8)**
IGF-I	2.0 (-1.1, 5.0)	0.8 (-1.9, 3.5)	0.5 (-3.9, 4.9)	0.3 (-4.1, 4.7)	-10.6 (-24.3, 3.0)	-1.6 (-11.7, 8.5)
IGF-I:IGFBP-3 molar ratio	2.2 (-1.1, 5.5)	-0.2 (-3.1, 2.7)	0.7 (-4.0, 5.5)	0.0 (-4.7, 4.8)	-14.3 (-28.7, 0.1)*	5.0 (-6.3, 16.3)
IGF-II	-0.9 (-4.3, 2.4)	-1.0 (-4.1, 2.0)	-2.6 (-7.5, 2.2)	-2.8 (-7.6, 2.0)	-1.1 (-18.2, 16.0)	0.0 (-12.0, 12.0)
IGF-II:IGFBP-3 molar ratio	-1.2 (-4.7, 2.4)	-2.7 (-5.8, 0.4)	-2.8 (-8.0, 2.4)	-3.7 (-8.8, 1.4)	-4.3 (-22.4, 13.8)	9.7 (-3.8, 23.2)
IGFBP-3	0.2 (-4.2, 4.5)	2.3 (-2.0, 6.5)	-0.3 (-6.8, 6.2)	-0.6 (-6.1, 7.3)	4.3 (-17.7, 26.4)	-12.7 (-27.1, 1.7)*

Statistics presented as difference (95% confidence interval). IGF, insulin-like growth factor; IGFBP, IGF binding protein; SHBG, sex hormone binding globulin. ^a^Adjusted for age at blood collection and age at mammography, batch, and ethnic group. ^b^Adjusted for age at blood collection and age at mammography, batch, ethnic group, and body mass index. ^c^Arithmetic scale, leptin was not log transformed. *P < 0.1, **P < 0.05, ***P < 0.01, ****P < 0.001.

Women with higher prolactin levels had higher mean percentage mammographic density, both before and after adjusting for BMI. Twofold-higher prolactin levels were associated with 2.2% higher percent density (95% CI: -0.1, 4.4) (borderline statistically significant). This association resulted from both a positive association with the area of dense tissue and an inverse association with the nondense area. There was no evidence that this, or any other, association differed between the two ethnic groups.

There was no evidence of an association of either IGF-I, IGF-II or IGFBP-3 (or their molar ratios) with any of the measures of mammographic density (relative or absolute) whether or not BMI was adjusted for.

**Figure 1:**
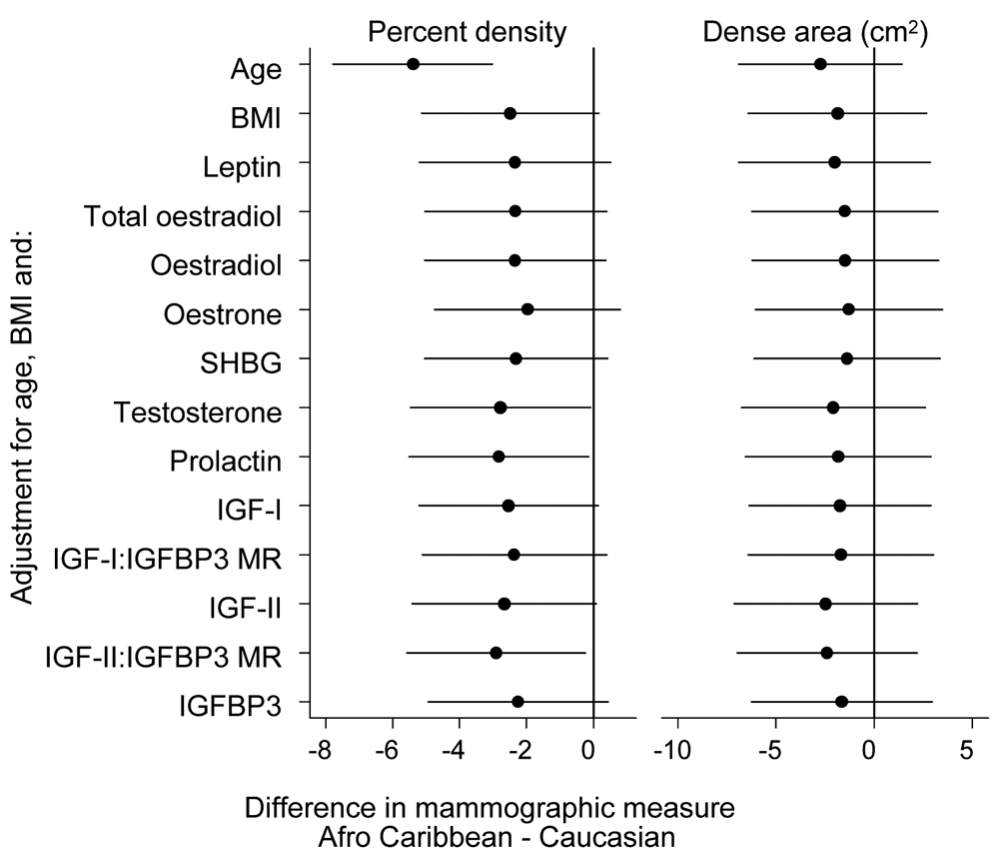
Ethnic differences in percentage mammographic density and dense area. Ethnic differences in percentage mammographic density (%) and dense area between Afro-Caribbean and Caucasian women, after adjusting for age, body mass index (BMI) and each plasma biomarker separately. IGF, insulin-like growth factor; IGFBP, IGF binding protein; MR, molar ratios; SHBG, sex hormone binding globulin.

Returning to ethnic differences in mammographic density, the age-adjusted mean percentage mammographic density was lower in Afro-Caribbean women than in Caucasian women, by 5.4% (95% CI: 3.0, 7.8). This difference was greatly attenuated upon adjustment for BMI (Table 2 and Figure 1) and further adjustment for blood biomarkers one at a time did not greatly affect this estimate, which was only attenuated to the null after controlling for reproductive breast cancer risk factors. These findings are not surprising given that the biomarkers that revealed ethnic differences (oestrogens and IGFs) were not associated with percentage mammographic density, and given that prolactin, which had a borderline positive association with percent density, exhibited no ethnic differences.

## Discussion

In this study of postmenopausal Caucasian and Afro-Caribbean women, higher levels of leptin, oestradiol (total and free), oestrone and testosterone were associated with lower percentage mammographic density, resulting from strong positive associations with the fatty area of the breast and no associations with dense area. The associations with percent density were attenuated to the null once BMI had been controlled for. Separate analyses of the effects on dense area, nondense area and percent density were informative and are particularly important when BMI is a strong confounder, as in the case of sex hormones that are adipose-derived in postmenopausal women. Determinants of percentage breast density that arise from an impact on the area of dense tissue, with or without a simultaneous effect on the area of nondense tissue, are more likely to be the relevant factors for breast cancer aetiology as the dense area represents stromal and epithelial tissue where breast cancers arise. Such factors may act through stromal and epithelial proliferation or breast involution. IGFs were not associated with any components of mammographic density either before or after adjusting for BMI. There was borderline statistically significant evidence that lower oestrone and higher prolactin levels were associated with higher percentage mammographic density. These biomarkers did not differ between ethnic groups, however, so they did not contribute to ethnic differences in percent density.

These results are largely consistent with those from previous studies of postmenopausal women. For oestradiol (total or free) and oestrone, inverse associations with percent density were attenuated upon adjustment for a measure of body size (BMI or waist circumference) in several previous studies [10,16-18], but with some exceptions. Aiello and colleagues found that inverse associations of oestrone and oestradiol (total and free) remained after adjustment for percentage body fat, but that this association was restricted to former hormone therapy users (all women were not taking hormone therapy at the time of blood/mammogram) – especially amongst women with hormone therapy use within the past 5 years, suggesting a long-lasting or residual effect of the exogenous hormones [10]. In a study by Boyd and colleagues, an inverse association of free oestradiol with both percent density and absolute dense area persisted after adjusting for waist circumference [11]. The only study to have found positive associations of oestrogens (oestrone and oestradiol) with percent density after BMI adjustment is that of Greendale and colleagues [12]. Our finding of a negative association with oestrone is surprising, but was only borderline statistically significant and may be a false positive finding.

These findings do not, however, rule out the possibility that breast density is affected by sex steroids. Most of the studies that have examined these associations have been cross-sectional in nature and have been carried out in postmenopausal women, whereas higher premenopausal levels of sex steroids are more likely to capture the aetiologically relevant exposure period. Lower mammographic density in more parous women, in older women and after the menopausal transition are suggestive of a hormonal basis to density. Furthermore, the striking parallels between the age profiles of percentage mammographic density, several sex steroids and Pike and colleagues' proposed model for the rate of breast tissue ageing [19] may result from common underlying biological processes. Studies of sex hormones in relation to percent density at premenopausal ages are needed, but are hampered by the unavailability of routinely conducted mammography at younger ages as well as by the difficulties in capturing a woman's average exposures to oestrogens given the large changes in hormonal levels across the menstrual cycle.

Neither IGF-I, IGF-II or their binding proteins were related to density; therefore, although their mean levels differed greatly by ethnicity, these factors did not explain ethnic differentials in percentage or absolute mammographic density. These findings are in agreement with several findings in postmenopausal women of null associations [10,20-23], although one study has reported a positive association [24]. As IGF-I is a breast cancer risk factor only at premenopausal ages, null associations with mammographic density in postmenopausal women may not be unexpected. IGF-I may still be a determinant of mammographic density at premenopausal ages, however, since positive associations have been observed in younger women [25] and IGF SNPs were reported to be associated with percent density irrespective of menopausal status [26].

Previous investigations into the effect of prolactin on mammographic density have been inconsistent – two found positive associations [11,27] and two were null [16,28]. Our findings do not help greatly to clarify this association, as we observed a weak positive association that was only significant at the 10% level. The circadian rhythm of prolactin, however, would give rise to large measurement error, reduced power and attenuation of regression slopes if a relationship truly exists. Although prolactin's role in breast development and lactation is well known, few determinants of this hormone other than parity are known [29]. It is plausible that mammographic density is on the causal pathway to breast cancer, as prolactin increases mitosis in the breast [28]. Further research into the prolactin–mammographic density association as well as the factors affecting prolactin levels are warranted in order to further clarify these associations.

The present study benefited from an ethnically diverse study population whose varied lifestyles give rise to very heterogeneous biomarker distributions in which, if associations exist, there was a wider range of exposures and outcomes within which they could be detected. The women were from a population-based screening programme and are representative of their ethnic groups [9]. We do not know the characteristics of the nonresponders, but it is possible that if factors associated with nonresponse (such as socioeconomic status) are also associated with breast density or biomarker levels, then the differential response rates by ethnic group might have biased ethnic differences in these levels. Associations between biomarkers and mammographic density, however – the focus of this paper – will not be affected as the observed associations are internal to the group of participating women.

Ethnic diversity included greatly differing BMI distributions, which, in the context of mammographic density, act as strong confounders. Coupled with this, adipose tissue is a major source of oestrogens in postmenopausal women, so adjustment for BMI is essential to disentangle any hormone – density associations that are not due to BMI. There remains the possibility of residual confounding as BMI was calculated from self-reported height and weight. Measurements at both the time of mammography and blood collection would have been more suitable. Measurement error may also attenuate results, especially for oestrone and prolactin that were less repeatable; however, error in the mammographic measurements was reduced through the use of average values across four films. The lag time between mammography and blood collection (approximately 1.7 years later) was not ideal and may have diluted findings, especially if hormone levels changed at different rates (for example, between younger and older postmenopausal women), but for the majority of women changes in hormone levels at these ages are likely to be small. The study was conducted in this fashion so as not to interfere with breast screening uptake. We did not adjust for the increased chance of false positive results from examining many exposures but, as the findings are essentially null, correction for multiple testing would not have changed the overall interpretation.

Cumulus-derived measures of breast density are area based and are a somewhat crude simplification of the three-dimensional breast. Two women with the same dense area may have very different volumes of dense tissue if the breast thickness, breast size and degree of breast compression differ. Given the lack of a validated volumetric approach to breast density measurement that can be applied retrospectively to digitised films, however, the use of Cumulus, a well-established method, was considered the best option currently available.

Lower breast cancer incidence rates, as reflected in marginally lower percentage mammographic density, in Afro-Caribbean women were not explained by levels of sex hormones, IGFs or prolactin as measured at a single time point postmenopausally. This measure, however, may not capture differences at younger ages or across the life-course. Lower density and breast cancer incidence rates could still be due to lower cumulative exposure to oestrogens in particular. At premenopausal ages, higher BMI is associated with lower oestrogen levels, and, combined with a later menarche and higher parity, total exposure to oestrogens at premenopausal ages is likely to be much lower in Afro-Caribbean women compared with Caucasian women. We have not been able to examine hormone–density associations at these ages as screening by mammography is only available to women aged 50 years and older in the UK.

## Conclusions

Mammographic density has one of the strongest associations with breast cancer risk, but the biological mechanism relating it to breast cancer is not clear. Greater density must result from greater rates of stromal proliferation, epithelial proliferation and/or slower rates of involution [30]. The chemical markers or processes that may influence these rates, and thus density, are not well identified.

Studies of first-generation migrants, as shown here, provide useful populations in which heterogeneity can be captured. Herein, despite large differences in breast cancer biomarkers (sex steroid and IGFs) between Afro-Caribbean women and Caucasian women, we did not observe associations of these biomarkers with postmenopausal mammographic density after controlling for the large confounding effect of BMI. The possibility remains, however, that mammographic density is influenced by these hormones, but that the critical period of exposure is when their levels are much higher, at premenopausal ages.

## Abbreviations

BMI: body mass index; CI: confidence interval; ELISA: enzyme-linked immunosorbent assay; IGF: insulin-like growth factor; IGFBP: insulin-like growth factor binding protein; SHBG: sex-hormone binding globulin; SNP: single nucleotide polymorphism.

## Competing interests

The authors declare that they have no competing interests.

## Authors' contributions

VAM coordinated data collection, carried out all statistical analysis and wrote the first draft of the paper. NJ, CP and BC participated in sample processing, MD and EF conducted sex steroid assays, JMH conducted IGF assays, SJV and NMP oversaw participant recruitment. IdSS participated in conceiving the study and in its design. All authors contributed to the analysis strategy, interpretation of results and writing of the paper. All authors read and approved the final manuscript.
